# S242. INTERNALIZED STIGMA AND ITS ASSOCIATION WITH CLINICAL SYMPTOMS AND PREMORBID ADJUSTMENT IN STABLE SCHIZOPHRENIA

**DOI:** 10.1093/schbul/sby018.1029

**Published:** 2018-04-01

**Authors:** Silvia Pardeller, Beatrice Frajo-Apor, Georg Kemmler, Annasara Meola, Fabienne Wartelsteiner, Wolfgang Fleischhacker, Alex Hofer

**Affiliations:** 1 Medical University Innsbruck; 2 University School of Medicine Federico II

## Abstract

**Background:**

Previous studies have shown that internalized stigma, i.e., the inner subjective experience of stigma resulting from applying negative stereotypes and stigmatizing attitudes to oneself, may impact negatively on schizophrenia patients’ quality of life, hope, and self-esteem and hinder the recovery process. The aim of the current study was to investigate to what extent patients’ internalized stigma correlates with both clinical symptoms and premorbid adjustment.

**Methods:**

We recruited patients with schizophrenia on an out-patient basis. Diagnoses were confirmed with the Mini International Neuropsychiatric Interview (M.I.N.I.). Internalized stigma was assessed by the Internalized Stigma of Mental Illness (ISMI) scale. Psychopathology was assessed by the Positive and Negative Syndrome Scale (PANSS), which was divided into five factors according to Wallwork et al.: positive, negative, disorganized/concrete, excited, and depressed. In addition, the Premorbid Adjustment Scale (PAS) was used, which covers two discrete areas of functioning: school functioning (scholastic performance and adaption to school) and social functioning (sociability and withdrawal, peer relationships, and capacity to establish sociosexual relationships).

**Results:**

So far, a total number of 69 patients (49.3 males, 50.7 females) with a mean age of 44.9 ± 10.4 years took part in this study. The mean duration of illness was 15.0 ± 10.5 years, the mean PANSS total score was 56.2 ± 16.9. With the exception of the Wallwork-factor “excited” (no significant relationship) clinical symptoms correlated negatively with the ISMI total score. Moreover, a negative association was seen between premorbid social functioning and internalized stigma, whereas no correlation was found between premorbid school functioning and the ISMI total score.

**Discussion:**

This study illustrates the complexity of factors that influence internalized stigma in stable outpatients with schizophrenia. Next to replicating earlier reports on positive associations between residual symptoms of the disorder and internalized stigma our observations point to the relevance of premorbid social functioning in this regard.

